# Communal Coping in Couples With Health Problems

**DOI:** 10.3389/fpsyg.2019.00398

**Published:** 2019-03-06

**Authors:** Kelly E. Rentscher

**Affiliations:** ^1^ Cousins Center for Psychoneuroimmunology, Semel Institute for Neuroscience and Human Behavior, University of California, Los Angeles, Los Angeles, CA, United States

**Keywords:** stress, coping, close relationships, couples, physical health, chronic illness

## Abstract

Prior to the 1990s, the predominant view of stress and coping defined stress as occurring when an individual perceives a situation as a challenge, threat, or loss and evaluates her capacity to respond based on her available resources. As an expansion of this intrapersonal perspective, the last 20 years have seen the emergence of two prominent interpersonal perspectives on stress and coping that account for the importance of social relationships in the coping process: the Systemic Transactional Model (STM) of dyadic coping and communal coping. In this article, I outline these two perspectives and highlight their points of convergence and divergence. I propose that one difference between the models is that communal coping involves an explicit focus on a communal or shared appraisal process, in which relationship partners view a problem or stressor as “ours” rather than “yours” or “mine.” I review existing methods for assessing communal coping (e.g., self-report, language use, behavioral observation) across laboratory, intervention, and real-world settings and summarize empirical evidence for the prognostic significance of communal coping for relationship and health functioning. I propose the utility of incorporating measurement of shared appraisal into future research on dyadic coping with stress, because of its potential to impact health through its influence on primary and secondary stress appraisal processes and physiological stress response systems. Finally, I outline biological and behavioral pathways through which communal coping may influence health as directions for future research.

## Introduction

Prior to the 1990s, the predominant view of stress and coping defined stress as occurring when an individual perceives a situation or an event as harmful or threatening by exceeding her available resources to address it. In their transactional theory of stress and coping, Lazarus and Folkman (1984) outlined a two-step appraisal process in which an individual first perceives a situation as a challenge or threat based on its ambiguity, controllability, and relevance to the self (primary appraisal), and then evaluates her capacity to respond to the situation based on the available resources (secondary appraisal). According to this theory, coping then involves the individual’s behavioral, cognitive, and/or social response in an effort to manage, reduce, or tolerate the demands of the situation. Lazarus and colleagues further categorized these coping responses or strategies as *problem-focused* when they aim to manage some aspect of the problem itself, or *emotion-focused* when they aim to manage the individual’s own emotional reaction to the problem. There is now an extensive literature on these appraisal and coping processes, which characterizes adaptive coping in terms of reductions in an individual’s psychological distress with great benefit for individual health and well-being. Over the last 20 years, the field has seen an expansion of this intrapersonal perspective on stress and coping, with the emergence of two prominent interpersonal coping perspectives that emphasize the importance of social relationships in stress appraisal and coping processes: the Systemic Transactional Model (STM) of dyadic coping (Bodenmann, 1995, 2005; Bodenmann et al., 2016) and communal coping (Lyons et al., 1998; Helgeson et al., 2018).

The paradigmatic shift toward an interpersonal perspective on stress and coping began in the early 1990s, when Coyne and colleagues conducted a series of studies with male patients who had experienced myocardial infarction and their female spouses. Importantly, the researchers’ observations while conducting this research led to findings that the wives’ own distress and coping efforts in response to the coronary event were correlated with their husbands’ coping responses, psychological adjustment, and health functioning following the event (Coyne and Smith, 1991, 1994; Fiske et al., 1991). Based on their findings and observations, the researchers concluded that myocardial infarction patients (and their spouses) are confronted with and manage health-related stressors in the context of their marital relationships, and that partners’ coping efforts also aim to manage and maintain aspects of their relationship during stressful periods. In addition to the established problem-focused and emotion-focused coping strategies, Coyne and Smith (1991) introduced the term *relationship-focused* coping, which refers to two interpersonal coping processes: (1) active engagement, where partners jointly discuss the situation, inquire about the other person’s feelings, and engage in collaborative problem-solving, and (2) protective buffering, where partners conceal their concerns, deny worries, or yield to each other to avoid conflict. These studies were some of the first to highlight the importance of close relationships and the role of marital partners in the coping process—not only as sources of support, but as active participants and collaborators in coping with stress and illness—that paved the way for the emergence of other interpersonal coping perspectives.

### The Systemic Transactional Model of Dyadic Coping

In the mid-1990s, Guy Bodenmann developed the STM of dyadic coping as a direct extension of Lazarus and Folkman’s transactional stress theory, in order to describe the processes through which romantic partners cope together with stress in the context of their relationship (Bodenmann, 1995, 2005). On a theoretical level, as reflected in the name, the STM of dyadic coping conceptualizes couples’ relationships as social systems in which romantic partners mutually influence each other; and by extension, stressful events affect both partners. On account of this mutual influence, coping with stressful events includes interactive processes that occur between partners in addition to primarily intrapersonal stress appraisal and coping processes. To differentiate these intra- and interpersonal stress appraisal and coping processes, the STM first outlines three types of stress: (1) *individual stress*, or stress that one partner is able to cope with alone without involving the other partner or asking for assistance, (2) *dyadic stress*, or individual stress that is unresolved because one partner is unable to successfully cope with it alone (i.e., due to ineffective appraisals, coping efforts, or resources) and the stress becomes relevant for the couple, and (3) *genuine dyadic stress*, or stress that directly concerns the couple as a unit (e.g., birth of a child, search for an apartment).

With respect to stress appraisal, the STM of dyadic coping extends Lazarus and Folkman’s model by proposing that individual partners engage in a primary appraisal process in which they evaluate the significance of a situation for their own well-being, their partner’s well-being, and the well-being of the relationship as a unit. In addition to this primary appraisal, the STM includes three additional appraisal processes, in which the individual partners (1) assess the other partner’s appraisal of the situation, (2) judge whether the other partner has realized his/her own appraisal, and (3) reevaluate and synthesize their own appraisal with their partner’s appraisal. The model further specifies that, after this reevaluation process, if both partners are in agreement a “common” or dyadic appraisal may result. The STM also expands upon Lazarus and Folkman’s definition of secondary appraisal by proposing that individual partners evaluate their own coping resources, their partner’s resources, and the resources of the relationship as a unit. In addition to this secondary appraisal, the STM includes two additional appraisal processes, in which individual partners (1) evaluate the secondary appraisal of the other partner, and (2) evaluate and synthesize their own appraisal with their partner’s appraisal.

Following individual appraisal of the stressful situation, the STM of dyadic coping outlines a stress communication process in which one partner shares his appraisal with the other partner, who interprets the partner’s communication and responds with some form of dyadic coping, that could range from taking action to ignoring the communication (Bodenmann, 2005). Importantly, one assumption of the STM is that partners engage in individual efforts as their first attempts at coping, and then engage in dyadic coping when the individual efforts are unsuccessful. With respect to coping responses, the STM adopts Lazarus (1980) and Lazarus and Folkman’s (1984) definitions of problem-focused and emotion-focused coping, but further defines dyadic coping as involving *all efforts* of one or both partners to manage stressful situations that affect one or both partners, in order to restore balance to the individual partners and to the relationship as a unit. The STM outlines three forms of dyadic coping: (1) *common coping*, where both partners attempt to manage a stressful situation together (e.g., through joint discussion or searching for information, mutual affection, common relaxation activities), (2) *supportive coping*, where one partner provides assistance to the other partner, and (3) *delegated coping*, where one partner requests that the other partner manage the stressful situation on account of the partner’s competency, resources, or experience.

In subsequent theory updates, Bodenmann (2005) and Bodenmann et al. (2016) expanded the STM to include both positive and negative forms of these dyadic coping processes, with positive forms comprising emotion- and problem-focused common, emotion- and problem-focused supportive, and delegated coping, and negative forms comprising hostile, ambivalent, and superficial coping. Bodenmann et al. (2016) further elaborated that common dyadic coping is expected to occur when a problem or stressful situation affects both partners, and when the partners perceive that their own personal resources may contribute to the coping process. Recently, research has also examined potential antecedents (e.g., communal goals as motivating factors) and consequences (e.g., increased sense of “*we*-ness” in the partners) of the dyadic coping process.

### Communal Coping

The paradigmatic shift that occurred in the 1990s also influenced Lyons et al. (1998) to develop an interpersonal coping perspective called communal coping, which emphasized the embeddedness of individuals within social relationships and the importance of interpersonal processes in coping with stressful life events. Based on interpersonal systems theory, the communal coping perspective conceptualizes couples and other social units (e.g., families, communities) as dynamic systems in which any change in one partner naturally affects the other partner, and affects the relationship as a whole. Lyons et al. (1998) argued that, because of the inherent interconnectedness between relationship partners, the distinction between intrapersonal and interpersonal stress appraisal and coping processes becomes superficial, as individual partners are simultaneously influenced by and consider the effects of a situation on their partners and relationships even when they are conceivably physically “alone.”

As an expansion of the relationship-focused coping—and active engagement, in particular—the communal coping perspective outlines a two-step appraisal and coping process. Specifically, communal coping occurs when one or both partners in a couple or other social unit (1) view a problem or stressful situation as “ours” (communal appraisal) rather than “yours” or “mine” (individualistic appraisal), (2) communicate about the stressful situation, including the details and meaning of the situation, and (3) engage in collaborative problem solving in which partners share responsibility for addressing the situation (communal action). The communal coping perspective further specifies that communication about the stressful situation may be verbal and/or nonverbal, and coping responses may involve conscious and/or unconscious action. As outlined above, communal coping includes two orthogonal dimensions—appraisal and action—that vary on a continuum from individualistic to communal/collaborative and form a four-quadrant model (Lyons et al., 1998, p. 586). Whereas one might locate the more traditional notion of social support in the lower-right quadrant, where partners work together to address a problem but still primarily view the problem as one person’s (individualistic appraisal, communal action), one would locate communal coping in the upper-right quadrant, where partners work together to address a problem *and* view the problem as shared (communal appraisal, communal action). Communal coping is therefore best distinguished from social support through its shared appraisal process, regardless of whether the problem originated as one partner’s problem or whether it produces similar consequences for both partners (Lyons et al., 1998). In addition, the upper left quadrant may best represent situations such as caregiving, in which partners view the problem as shared but one person assumes primary responsibility for addressing it (communal appraisal, individualistic action). For example, a woman whose partner has an illness that makes it difficult to care for himself might view a health-related problem as shared, yet assume primary responsibility for caregiving tasks. Finally, individual coping is located in the lower left quadrant, where partners view the problem as one person’s and engage in solo efforts to address it.

In reference to Lazarus and Folkman’s (1984) transactional theory of stress and coping, engaging in communal coping is likely to influence primary stress appraisal processes (Rentscher, 2017; Helgeson et al., 2018). Specifically, regardless of whether the problem originated as one person’s, the partners appraise the problem as a challenge or threat with relevance to the couple *with little distinction between the self and the partner*. Communal coping is also likely to influence secondary stress appraisal processes, such that when partners assess their available coping resources they *explicitly or implicitly* draw on their partner’s available resources in addition to their own. This “doubling” of available resources provides greater diversity of resources and a more effective set of coping strategies that may render communal coping more effective at buffering stress than social support, in which the partners’ resources may be available if needed but are still provided from one person to the other rather than pooled or shared (Lyons et al., 1998). It is through these primary and secondary appraisal processes that the magnitude of a problem may be reduced and the stress response buffered. Finally, communal coping involves active coping responses characterized by collaborative problem solving and coordinated efforts to reduce the impact of the stressor.

In a recent theory update and review, Helgeson et al. (2018) proposed expanding the communal coping model in several ways based on their work with couples coping with type 2 diabetes. First, the authors proposed that among couples in which one partner has a chronic illness, partners’ coping efforts are primarily aimed at improving the health and well-being of the identified patient. Second, whereas Lyons et al. (1998) posited that communal coping occurs when *one or both* partners view a problem as shared, the authors argued that in this context the benefits of communal coping are strongest when both partners adopt a communal or shared illness appraisal. Third, the authors proposed that supportive behaviors that one partner provides to the other that might otherwise be characterized as (unidirectional) social support may be construed as collaborative actions when partners hold a shared appraisal. Preliminary evidence for this hypothesis comes from a recent study of couples with type 2 diabetes in which shared illness appraisals moderated the association between spousal emotional support and effective patient self-management of the illness. Finally, the authors proposed a conceptual model that also outlines potential (1) antecedents of communal coping (e.g., relationship quality, nature of the illness) and (2) mechanisms (e.g., self-efficacy, reduced stress appraisals) through which communal coping may influence chronic illness adjustment.

### Comparison of Interpersonal Coping Perspectives

The STM of dyadic coping and communal coping have several important points of convergence and divergence. With respect to points of convergence, both models (1) adopt a systemic theoretical approach and emphasize interdependence between relationship partners, (2) identify relationship quality or satisfaction as an antecedent of interpersonal coping, (3) include a dyadic form of stress appraisal in which one or both partners view a problem or stressful situation as relevant to the relationship, and (4) describe a collaborative coping response in which partners discuss the problem and engage in coping efforts together. Of the two models, the communal coping perspective is narrower in its scope. Although the model also describes individual, caretaking, and social support forms of coping, it defines communal coping as a specific process in which one or both partners view a problem as shared and engage in collaborative problem solving to address it. In comparison, the STM of dyadic coping is broader and more detailed in its description of a range of stress appraisal and coping responses that may occur within and between partners as the coping process unfolds.

In addition to these similarities, the points of divergence between the models suggest areas for development in future research. The first point concerns the type of stress and outcome of interest that have historically been the focus of each of the respective models. The STM of dyadic coping was originally developed to investigate everyday stressors (e.g., daily hassles) in more normative, non-clinical community samples of couples and the majority of research has focused on the effects of dyadic coping on relationship satisfaction (Bodenmann et al., 2016). Interestingly, more recent research has applied the STM of dyadic coping to health problems such as cancer (Badr et al., 2010; Manne et al., 2014; Regan et al., 2014; Rottmann et al., 2015), type 2 diabetes (Johnson et al., 2013), and chronic obstructive pulmonary disease (Meier et al., 2011). By contrast, although the communal coping perspective was originally developed to investigate a broad array of stressful life events and social units (e.g., couples, families), the majority of research has focused on clinical samples of couples with health problems such as congestive heart failure, type 2 diabetes, and substance use disorders (see subsequent section for review). Moreover, the outcomes of interest in these studies have ranged from chronic illness adjustment, adherence to the medical regimen, health behavior change, and disease course (e.g., symptom severity).

On a theoretical level, a second difference between the models involves the emphasis each model places on shared appraisal as the primary response to stress (Rentscher, 2017; Helgeson et al., 2018). The STM specifies that partners engage in individual coping processes as their first coping attempts and turn to dyadic coping processes if individual efforts are unsuccessful, whereas communal coping emphasizes that partners can engage in shared appraisal or collaborative action at the very initiation of a problem regardless of whether the problem originated as one person’s. Although the STM outlined that the partners can arrive at a dyadic appraisal of a problem (which recent papers have also referred to as “*we*-stress” or “*we*-disease,” depending on the nature of the stressor; Bodenmann et al., 2016), the model does not specify that the partners may adopt a dyadic appraisal from the initiation of an individual or dyadic stressor. In this way, the emphasis on the initial appraisal of a problem as “ours” is a unique aspect of the communal coping perspective; however, it is important to note that the primacy of shared appraisals is a theoretical concept that remains to be investigated empirically.

Emerging neuroscience research based on Social Baseline Theory provides preliminary evidence in support of the primacy of shared appraisals. Social Baseline Theory, developed by James Coan, posits that social contact and relatedness—rather than isolation and aloneness—are the natural or “baseline” conditions of the human brain, and that individuals’ proximity to and interaction with others serves to regulate important aspects of the neural response to threat (Beckes and Coan, 2011; Coan and Maresh, 2014; Coan and Sbarra, 2015). To test this idea, Coan et al. (2006) conducted a functional imaging study in which women were exposed to threat of electric shock while holding either their spouse’s hand or a stranger’s hand. Women holding their spouse’s hand showed greater attenuation in activation of brain regions associated with threat responding compared to those holding a stranger’s hand, and women with higher marital quality showed larger reductions in the threat-responsive brain regions. In a recent replication and extension of this study, individuals exposed to threat of shock while holding hands with a close other (spouse, dating partner, friend) showed greater attenuations in several threat-responsive brain regions compared to those holding hands with a stranger or being alone in the scanner (Coan et al., 2017). Moreover, individuals reporting greater social support showed larger attenuations when holding hands with a close other. Adopting a similar paradigm, Beckes et al. (2013) assessed individuals’ neural activation in response to threat of electric shock themselves or observing a threat of shock to a friend or stranger. Neural activation in response to threat to the self was significantly correlated with threat to a friend in several threat-response regions, but less correlated with threat to a stranger. Furthermore, individuals who reported higher scores on the Inclusion of Other in Self (IOS; Aron et al., 1992) scale, a measure of self-other overlap, showed increased activation for threat to a friend but not to a stranger. Although this study did not involve a romantic partner, findings suggest a blurred distinction between self and close others at the neural level. Together, these studies suggest that relationships marked by interdependence and closeness may influence partners’ stress response at the initial appraisal of a stressor, and by extension, that partners may engage in shared appraisal and coping processes as a baseline, or primary response to stress; however, this hypothesis remains to be tested.

Finally, and related to the previous point, a third difference concerns the availability of well-developed measures to assess the stress appraisal and coping processes each of the models propose. With respect to the STM, the Dyadic Coping Inventory (DCI; Bodenmann, 2008) has been translated into multiple languages and is widely adopted in research on interpersonal coping. Although the DCI reliably measures the various forms of positive and negative dyadic coping responses, well-developed measures of the stress appraisal processes proposed in the model have not yet been developed. Recent advances in research on communal coping have included newly developed and validated measures for assessing both shared appraisal and collaborative action; however, some of the measures are early in their development and will benefit from additional validation in future studies. In light of recent expansions to the STM of dyadic coping that have described dyadic appraisals as “*we*-stress” or “*we*-disease” (adapted from Kayser et al., 2007), the communal coping perspective offers theoretically-consistent and validated methods for assessing shared appraisal processes that may also be of benefit to future research based on the STM and other dyadic coping perspectives.

As a more extensive review of the empirical literature on the STM of dyadic coping goes beyond the scope of this paper (see Falconier et al., 2016, for an detailed review), the remainder of this paper focuses on the contributions of the communal coping perspective to the literature with respect to advances in measurement, associated empirical findings, and an explicit emphasis on shared appraisal processes (in addition to couple collaboration) in the context of coping with health problems.

## Approaches to Measuring Communal Coping

### Self-Report Measures

Rohrbaugh et al. (2008) were the first to develop a self-report measure of communal coping in a study of 60 couples in which one partner had congestive heart failure. The measure is comprised of one item related to shared appraisal (“When you think about problems related to your/your partner’s heart condition, to what extent do you view those as ‘our problem’ (shared by you and your spouse equally) or mainly your own problem?”), and one item related to collaborative action (“When a problem related to your/your partner’s heart condition arises, to what extent do you and your partner work together to solve it?”) that partners rated on a 5-point scale. In this sample, the two items were moderately correlated for both patients and spouses, so the authors averaged them to form a communal coping score for each partner. Patient and spouse communal coping scores were correlated with patients’ use of first-person plural pronouns (*we*-talk; described below), providing some evidence of external validity. Surprisingly, self-reported communal coping did not relate to patient health outcomes in this study. On average, partners reported high levels of communal coping (*M_patients_* = 4.1, *M_spouses_* = 4.6 out of 5); therefore, associations may have been constrained by a restricted range of scores on the scale.

In a recent study of 123 couples in which one partner had type 2 diabetes, Helgeson et al. (2018) expanded the Rohrbaugh et al. (2008) measure by adding three additional items. The five-item scale is comprised of two items related to shared appraisal (e.g., “When you think about problems related to your diabetes, to what extent do you view this as “our problem” [shared by you and your spouse equally] or mainly your own problem?”) and three items related to collaborative action (e.g., “When a problem related to your diabetes arises, how much do you and your spouse work together to solve it?”). The authors averaged the items to form a communal coping score for each partner. Although psychometric information for the scale is not available, partner communal coping scores were significantly associated with relationship well-being, providing some evidence of external validity.

In the same sample of couples coping with diabetes, Helgeson et al. (2018) also developed a daily diary version of the scale that includes one item related to appraisal (e.g., “When you thought about diabetes today, did you view diabetes as “our problem” (shared equally by you and your partner) or mainly your own problem?”) and one item related to action (e.g., “How much did you and your spouse work together to take care of diabetes?”). Partners separately completed the two items at the end of each day for 14 consecutive days. The two items were significantly correlated for both patients and spouses, so the authors averaged them to form a daily communal coping score for each partner. Patient and spouse communal coping scores were also significantly correlated on a daily basis (Zajdel et al., 2018). Psychometric information for the scale, including within-person reliability estimates, is not yet available. Finally, in the same sample of couples coping with diabetes, Helgeson et al. (2017) adapted the IOS (Aron et al., 1992) scale to create a single-item measure of communal coping. Whereas the original IOS scale was comprised of seven concentric circles that overlap to various degrees and individual partners select the pair of circles that best represents their relationship (from no overlap to complete overlap), the adapted version of the IOS asked partners to select the pair of circles that best represents how the couple has *coped* with the diabetes diagnosis (also ranging from no overlap to complete overlap). Patients’ scores on this adapted version of the IOS correlated significantly with relationship quality, providing some evidence of external validity in this sample of couples coping with diabetes.

### Language Measures

Rohrbaugh et al. (2008) were the first to investigate couples’ first-person plural pronoun use (*we*-talk) as an unobtrusive, linguistic indicator of communal coping in the context of health-related communication. Of note, this study extended a sizeable body of research on *we*-talk in the context of couple communication as a linguistic marker of relational *we*-ness, that has been associated with greater positive and fewer negative interaction behaviors, more effective problem solving, and lower physiological activation during conflict (Simmons et al., 2005; Seider et al., 2009; Williams-Baucom et al., 2010). In this study, 60 couples in which one partner had congestive heart failure participated in a conjoint, coping-focused interview. To derive language measures, research assistants observed the video-recorded interviews and prepared verbatim transcripts of patient and spouse speech. Researchers submitted the transcripts to Linguistic Inquiry and Word Count (LIWC; Pennebaker et al., 2015), which extracted first-person plural (*we, us, our*) and first-person singular (*I, me, my; I-*talk) pronoun use scores as proportions of each partner’s total word count. The authors then created a *we/I*-ratio score to represent the proportion of total first-person pronouns that was plural rather than singular. *We*-talk and *I*-talk scores were significantly inversely correlated for patients and marginally inversely correlated for spouses. Partner *we/I*-ratio scores were not significantly correlated. Furthermore, *we/I*-ratios were significantly associated with scores on the two-item communal coping scale for patients and relationship quality for both partners, providing some evidence of external validity.

In a sample of 70 couples coping with diabetes, Helgeson et al. (2017) also investigated partners’ first-person plural pronoun use as a proportion of each person’s total pronoun (first-, second-, and third-person pronouns) use during separate interviews with patients and spouses about how they had coped with diabetes. Patient and spouse *we*-talk proportion scores were significantly correlated. Somewhat surprisingly, however, partner *we*-talk scores were not significantly correlated with scores on the adapted IOS as a measure of communal coping.

### Observational Measures

Helgeson and colleagues were the first to develop a global observational measure of communal coping in a sample of 123 couples in which one partner had type 2 diabetes. Research assistants observed videotaped interaction tasks in which the couples discussed a diabetes-related stressor and rated communal coping behavior for each partner. The measure includes a single, global item with a five-point scale ranging from 1 (low communal coping) to 5 (high communal coping). The measure instructs observers to rate the extent to which the patient or spouse views the current stressor as a joint problem based on a careful review of the whole interaction. The scale demonstrated excellent interrater reliability, with intraclass correlation coefficients (ICCs) ranging from 0.79 to 0.80. Partner observational communal coping scores were significantly correlated with scores on the five-item communal coping self-report scale and with *we*-talk during individual coping-focused interviews, providing evidence of external validity. In addition, partner observational communal coping scores were significantly correlated (Van Vleet and Helgeson, 2016; Van Vleet et al., 2018).

Recently, Rentscher and colleagues developed an expanded, four-item observational measure of communal coping designed to capture therapeutic change processes in a study of 56 couples participating in couple-focused interventions for health problems. The measure is comprised of four items that assess the shared appraisal dimension (e.g., “To what degree does the patient/spouse view the problem as one individual’s (“my” or “your”) problem or a shared (“our”) problem?”), the collaborative action dimension of communal coping, (“To what degree does the patient/spouse deal with the problem by working alone or working together as a team?”), and a third *we*-ness dimension (“To what degree does the patient/spouse show a sense of independence/separateness or togetherness/*we*-ness as part of the couple?”) designed to measure the extent to which each partner shows a sense of togetherness or interdependence as part of the couple. Trained raters observed the video-recorded therapy sessions and rated each partner in 1-min micro-segments using a nine-point bipolar scale ranging from −4 (e.g., individual problem) to +4 (e.g., shared problem). The scale demonstrated strong interrater reliability across the 1-min segments (ICCs ranged from 0.54 to 0.82) and excellent internal consistency (Chronbach αs ranged from 0.93 to 0.95); therefore, the four items were averaged across raters and items to form a single observational communal coping score for each partner. Partner observational communal coping scores were significantly correlated with *we*-talk during the therapy sessions, providing evidence of external validity. In addition, partner observational communal coping scores were significantly correlated (Rentscher, 2017; Rentscher et al., 2017, 2018).

## Summary of Empirical Findings on Communal Coping

### Self-Report Findings

In a sample of 60 couples in which one partner had congestive heart failure, partner scores on the two-item communal coping scale were significantly associated with relationship quality (Rohrbaugh et al., 2008); however, they did not predict changes in patient heart failure symptoms or general health functioning over a 6-month follow-up period. In a sample of 123 couples coping with diabetes, Van Vleet and Helgeson (2016) investigated associations between the five-item communal coping scale and relationship functioning within an actor-partner interdependence model (APIM). Results revealed significant actor effects, suggesting that one’s own communal coping was associated with one’s own reported relationship well-being and more positive perceptions of one’s partner. In a separate analysis with this sample, patient reports of communal coping were also significantly associated with diabetes self-care (e.g., diet, exercise, medication adherence; Helgeson et al., 2018). The daily diary reports of communal coping were also investigated in an APIM framework, with a significant actor effect suggesting that higher levels of one’s own communal coping on a given day was associated with lower depressed and angry mood and higher happy mood that day (Zajdel et al., 2018). In addition, a significant partner effect suggested that higher levels of communal coping from one’s spouse on a given day is associated with one’s own happy mood that day. Finally, in a smaller sample of 70 couples coping with diabetes, patient scores on the adapted IOS were positively correlated with relationship quality, and partner scores were negatively correlated with spousal psychological distress (Helgeson et al., 2017).

### Language Findings

In a study of 60 couples in which one partner had congestive heart failure, patient and spouse w*e/I*-ratio scores derived from the coping-focused interview were significantly associated with relationship quality (Rohrbaugh et al., 2008). Interestingly, spouse *we/I*-ratio scores predicted patient relationship quality over and above patients’ own *we/I*-ratio scores, and *vice versa*. Somewhat surprisingly, patient and spouse pronoun use was not associated with measures of patient health status at baseline; however, spouse *we-*talk scores predicted positive changes in patients’ heart failure symptoms and general health functioning over a subsequent 6-month follow-up period. Follow-up analyses revealed that active *we*-talk (*we* vs. *us/our*) accounted for the association with heart failure symptom course. In a study of 75 couples in which one partner had breast cancer, spouse *we*-talk during a family coping-focused interview was significantly associated with marital quality and fewer patient depressive symptoms (Robbins et al., 2013). Finally, in a recent study of 70 couples coping with diabetes, spouse *we*-talk was associated with less patient psychological distress and greater diabetes self-care (e.g., diet, exercise, medication adherence; Helgeson et al., 2017).

In the first study of couples’ pronoun use during a clinical intervention, 20 couples in which one partner continued to smoke despite having heart or lung disease participated in a couple-focused smoking intervention (Rohrbaugh et al., 2012). Researchers derived pronoun measures from a pretreatment couple interaction task, as well as three 5-min segments sampled from two subsequent therapy sessions that were combined into one *we*-talk score for each partner. In this sample, *we*-talk over the course of the intervention was associated with relationship quality for patients but not spouses. In addition, spouse *we*-talk during the baseline interaction and increases in *we*-talk by both patients and spouses from pretreatment through the intervention predicted patients’ smoking cessation success 1 year following treatment. In a combined study of four clinical trials, 188 couples in which one person had an alcohol use disorder participated in a couple-based behavioral intervention for alcohol use (Hallgren and McCrady, 2016). Patient *we*-talk during the first therapy session predicted the percentage of abstinence days later in the treatment, and spouse *we*-talk during the first session predicted patients’ percentage of abstinence days 6 months following treatment. In another study, 33 couples in which one person had an alcohol use disorder participated in couple-focused interventions for alcohol use (Rentscher et al., 2015). Researchers derived pronoun measures from a pretreatment interaction task in which couples discussed the alcohol problem, as well as three 5-min segments sampled during three subsequent therapy sessions that were combined into one *we*-talk score for each partner. Spouse *we*-talk during the intervention (accounting for pretreatment *we*-talk) uniquely predicted successful treatment outcomes, especially when distinguishing active from passive (*we* vs. *us/our*) pronoun forms.

Interestingly, several of these studies report asymmetric (i.e., partner) effects, whereby spousal *we*-talk predicts patient health outcomes over and above the patient’s own pronoun use (Rohrbaugh et al., 2008, 2012; Rentscher et al., 2015; Robbins et al., 2013). Considering that each of these studies involved couples in which one partner was the identified patient, changes in communal coping by the spouse (e.g., viewing the patient’s health problem as their own, engaging in problem solving with the patient) may be particularly important in this context and therefore more predictive of outcomes than the patient’s own communal coping. It is important to note, however, that although most of the existing research has found positive effects of spousal communal coping on patient health, one study found that asymmetric patterns in couple *we/I-ratios*, characterized by more *we*-talk relative to *I*-talk by the spouse than the patient, was associated with problematic demand-withdraw interaction patterns, suggesting a potential boundary condition of adaptive communal coping—at least when partners are discrepant in their approach to (communal) coping (Rentscher et al., 2013).

Finally, in a combined sample of 56 couples participating in couple-focused interventions for health problems, Rentscher et al. (2017, 2018) aimed to investigate communal coping within an experimental medicine framework. Researchers derived pronoun measures from a single target session in which changes in communal coping were expected to occur. Both patients and spouses showed within-session increases in *we*-talk and deceases in *I*-talk following therapist implementation of a set of therapeutic techniques designed to activate communal coping, providing evidence of construct validity and suggesting successful engagement of communal coping as a therapeutic target in the couple-focused interventions. Although these studies did not directly assess the potential mechanisms that may account for increases in communal coping during the interventions, it is possible that the therapeutic techniques implemented by the therapist strengthened the partners’ shared appraisal by reinforcing a sense of cohesion or togetherness and shared identity as a couple. The techniques may have also strengthened collaboration by exploring how the partners worked together to successfully resolve difficulties in the past, increasing the time spent together, and focusing on couple communication about the health problem both during and outside of the therapy sessions (Rentscher, 2017).

### Observational Findings

In a sample of 123 couples in which one partner had type 2 diabetes, observational ratings of patient communal coping behavior were associated with improvements in diabetes self-care and decreases in diabetes-related distress 6 months later (Van Vleet et al., 2018). In addition, within an APIM, a significant actor effect suggested that one’s own communal coping behavior was associated with greater relationship quality (Van Vleet and Helgeson, 2016). In addition, in a combined sample of 56 couples participating in couple-focused interventions for health problems, Rentscher et al. (2017, 2018) also found that both partners showed within-session increases in observable communal coping behavior following therapist implementation of a set of techniques designed to activate communal coping, providing additional evidence of construct validity and successful engagement of communal coping as a therapeutic target in the couple-focused interventions.

### Summary

Over the past 10 years, a growing body of research has linked communal coping—particularly by the spouse—to better relationship functioning, adjustment to chronic illness, physical health outcomes, and health behavior change in couples coping with health problems. In addition, researchers have developed a variety of measures for assessing communal coping across laboratory, intervention, and real-world settings. Of these measures, behavioral (i.e., language, observational) assessments of communal coping have shown particularly strong potential as they reduce social desirability concerns inherent in self-report measures, especially for highly evaluative constructs such as communal coping. Indeed, in their recent review, Helgeson et al. (2018) reported that their observational measure of communal coping was the most predictive of psychological and behavioral outcomes in their sample of couples coping with type 2 diabetes. Language measures (e.g., *we*-talk) also have the advantage of serving as unobtrusive indicators of communal coping that have demonstrated strong predictive validity in couples coping with diverse health problems.

## Future Directions and Conclusions

**Figure 1:**
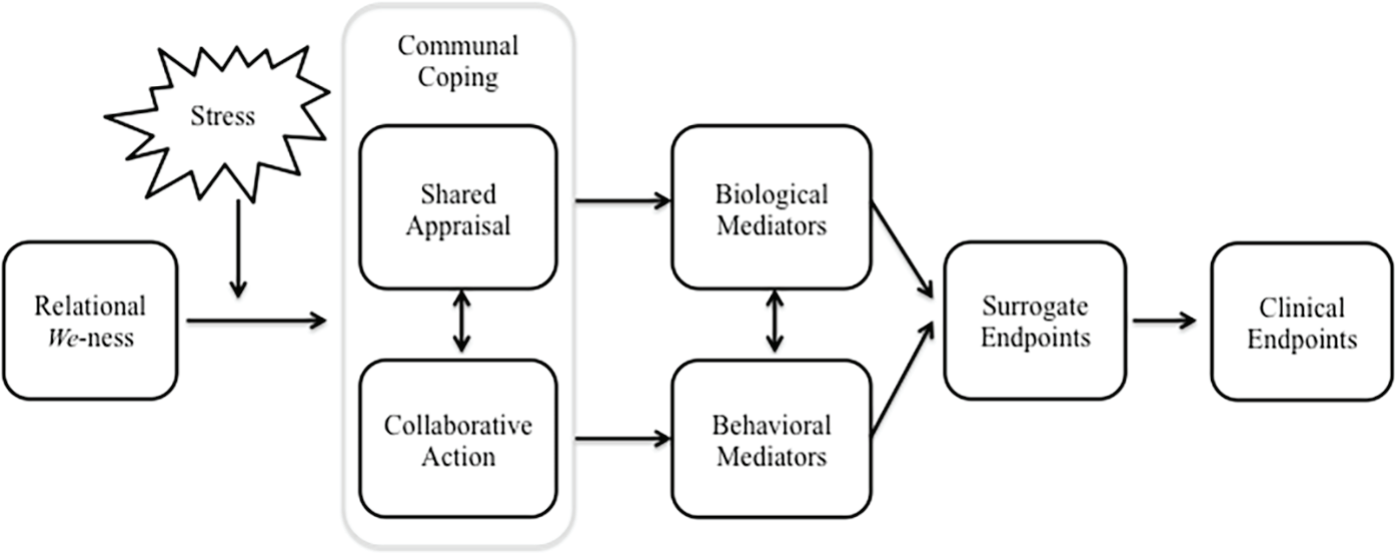
Conceptual model depicting potential pathways through which communal coping may affect health.

In their recent theory update and review, Helgeson et al. (2018) proposed a conceptual model that outlined the process through which communal coping influences adjustment to chronic illness. In this model, patient adjustment is defined to include psychological well-being, self-care behavior (e.g., adherence to the medical regimen), and physical health, and partner adjustment is defined in terms of psychological well-being. As a direction for future research, I propose an expansion of this conceptual model that includes (1) a delineation of the potential biological and behavioral pathways that may link communal coping to health outcomes, (2) a differentiation of the appraisal and action dimensions of communal coping, and (3) an emphasis on relational *we*-ness as a unique aspect of relationship quality and antecedent of communal coping. These directions for future research are detailed below and summarized in an integrated conceptual model (Figure 1), whereby in the face of stress, a couple’s sense of *we-*ness serves as a relational resource that translates into or activates a process of communal coping (i.e., shared appraisal, collaborative action), which in turn influences biological and behavioral pathways to affect health outcomes. This proposed conceptual expansion suggests several new directions for future research in this area.

### Biobehavioral Pathways Linking Communal Coping to Health Outcomes

Robles et al.’s (2014) meta-analytic review on marital quality and health provides a useful framework for identifying the biological and behavioral pathways through which relational constructs like communal coping might impact health, and clarifying how researchers can conceptualize health outcomes with greater specificity. Broadly, the authors outlined that marital quality (both support and strain) influences several psychological (social-cognitive and affective processes, psychopathology) and behavioral (health behavior) pathways, which then impact biological mediators, surrogate endpoints, and clinical endpoints. The authors defined clinical endpoints as subjective measures of health functioning (e.g., health-related quality of life, physical symptoms, pain severity, functional impairment), objective measures of health status (e.g., occurrence of a heart attack, hospitalization), and mortality. In comparison, surrogate endpoints are biomarkers that predict clinical endpoints but represent earlier events in the disease process, such as cholesterol levels or blood pressure predicting future cardiovascular disease endpoints (e.g., coronary artery disease). Finally, biological mediators are biomarkers that are not surrogate endpoints but represent allostatic and restorative processes in response to short term demands that contribute to longer term health outcomes (Robles and Carroll, 2011). Whereas allostatic processes involve dysregulation in cardiovascular, neuroendocrine (e.g., sympathetic-adrenal-medullary and hypothalamic–pituitary–adrenal axes), and/or immune (e.g., inflammation) systems, restorative processes are complementary and help restore these biological systems to their original state prior to the demand. 

In addition to the psychological mediators Helgeson et al. (2018) outlined, I propose expanding the model to include biological (e.g., cardiovascular, neuroendocrine, immune) and behavioral (e.g., health behavior) mediators, as well as surrogate and clinical endpoints based on Robles et al.’s (2014) model (Figure 1). To date, the majority of research on communal coping has investigated associations with health behavior and behavior change (e.g., adherence to the medical regimen, alcohol abstinence, smoking cessation) or clinical endpoints (e.g., heart failure symptom course, self-reported physical health), but the biological mechanisms remain untested. In addition, whereas most of the existing literature linking marital quality to biological mediators has focused exclusively on allostatic biological processes, less is known about how aspects of close relationships may influence restorative processes. Moreover, in a recent theoretical paper, Slatcher and Schoebi (2017) call for a greater emphasis on “marital strengths,” or the positive aspects of relationships that may have unique effects on these biobehavioral mechanisms and health outcomes (e.g., over and above the more negative aspects of relationships) and serve as a protective buffer of the negative effects of stress on health; an idea that is consistent with the conceptual model proposed here.

### Distinguishing the Appraisal and Action Dimensions of Communal Coping

Previous research has not been able to differentiate the appraisal and action dimensions of communal coping, as the dimensions have been highly correlated when assessed with self-report and observational scales. However, future research may benefit from further investigation in this regard (Helgeson et al., 2018; Rentscher et al., 2018), because although the two dimensions are related, they may have unique relevance to the biological and behavioral processes that may link communal coping to health outcomes.

First, the shared appraisal dimension may have a particular impact on biological mediators, because of its potential to influence primary and secondary stress appraisal processes, and therefore physiological stress response systems. Preliminary evidence in support of this hypothesis comes from the Coan et al. (2006) finding that under threat of shock, women who held their partner’s hand showed reduced activation of threat-related neural regions, and this attenuation was greater for women reporting higher marital satisfaction. Although not tested directly, differences in these neural regions may have “downstream” effects on key allostatic processes such as cardiovascular, neuroendocrine, and immune system activation. Second, in a study of non-clinical couples, Seider et al. (2009) found that *we*-talk by one’s partner during discussion of a relationship conflict was associated with lower cardiovascular arousal for oneself. Finally, in another study of non-clinical couples, Helgeson et al. (2016) found that individual partners who participated in an acute laboratory stressor had lower blood pressure and heart rate and faster physiological recovery when researchers framed the stressor as a shared (i.e., the responsibility of both partners) rather than an individual stressor. Importantly, each of these studies suggests that communal appraisals in the face of threat or challenge may lessen the impact on physiological stress response pathways.

There is also a sizeable literature on social support that suggests that the proposed biological mediators are plausible pathways that may link communal coping to clinical endpoints. In a seminal review, Uchino (2006) summarized a body of epidemiological research establishing reliable links between social support and mortality from cardiovascular disease, cancer, and infectious diseases, and growing evidence that social support may impact morbidity and mortality through cardiovascular, neuroendocrine, and immune pathways. As outlined previously, the primary difference between social support and communal coping is that communal coping involves the presence of a communal or shared appraisal process in which relational partners view a problem or stressful situation interdependently regardless of whether the problem originated as one person’s. That is, in the face of stress, social support may involve collaborative action in which partners work together to address a problem, but communal coping occurs when partners also view the problem as shared. Adopting a shared appraisal implies a degree of interdependence in the relationship that is likely to also influence both primary and secondary stress appraisals. Helgeson et al. (2018) further proposed that engaging in shared appraisals may also help partners view supportive behaviors—which are typically unidirectional, with one partner providing support to the other—as a collaborative effort to address the problem, thereby increasing the effectiveness of support behaviors. For these reasons, communal coping has the potential to confer comparable or even additional benefits for physical health through similar pathways as social support; however, this hypothesis remains to be tested in future research.

Second, the collaborative action dimension of communal coping may have a particular influence on behavioral mediators, as partners are likely to engage in health behaviors together and partner collaboration may also serve as a resource for health behavior change (Rohrbaugh, 2014). Preliminary evidence in support of this hypothesis comes from a meta-analysis of relationship factors that contribute to patient adherence to medical treatment. Although the meta-analysis did not directly examine collaboration, DiMatteo (2004) found that medical adherence was 1.7 times higher among patients with greater family cohesion and 1.5 times lower among patients with greater family conflict. In addition, individuals who reported greater closeness with their spouse were more successful in reducing their substance use over the course of individual treatment (Heinz et al., 2009). Finally, in recent studies of couple-focused intervention outlined in this review, increases in communal coping from pretreatment through the course of the intervention predicted successful patient smoking cessation and alcohol abstinence (Rohrbaugh et al., 2012; Rentscher et al., 2015). Together, these studies suggest that collaborative action to address a health problem or stressor may promote health behavior change in couples and serve as a behavioral target for interventions.

Given the potentially unique relevance of shared appraisal and collaborative action dimensions for the biological and behavioral processes that may link communal coping to health outcomes, it will be important for future research to distinguish these dimensions methodologically as well as conceptually. Although current measurement approaches have not be able to disentangle the dimensions, refinement of existing self-report, language, and observational measures may allow researchers to quantify potential differences. For example, self-report and observational instruments might be expanded to test several items for each dimension, and linguistic tools (e.g., machine learning) may be developed to detect patterns in the verbs that accompany instances of partner *we*-talk. For example, shared appraisal might be captured by phrases that include linking verbs such as “we are/seem/feel,” whereas collaborative action might be captured by phrases that include action verbs such as “we do/talk/eat.” Future research could also incorporate functional imaging to further investigate these dimensions at the neural level and ecological momentary assessment methods (e.g., the Electronically Activated Recorder; Mehl, 2017) to naturalistically observe these processes in daily life, extending investigations of communal coping beyond laboratory and therapy contexts and potentially increasing the range of observed behavior.

### Relational *We*-ness as an Antecedent of Communal Coping

Buehlman et al. (1992) were the first to conceptualize *we*-ness (versus separateness) in married couples as the extent to which the partners identified themselves as part of a couple rather than emphasizing their individuality or independence. The authors found that observational ratings of *we*-ness—which were largely based on the partners’ use of *we*-talk—were associated with greater positive and fewer negative emotional expressions during couple interactions. Since this original study, the majority of research has utilized computerized text analysis to unobtrusively measure partner *we*-talk as a linguistic marker of *we*-ness, finding that couples who engage in more *we*-talk also tend to engage in more positive (e.g., affectionate) and fewer negative (e.g., hostile) behaviors and solve problems more effectively (Simmons et al., 2005; Williams-Baucom et al., 2010). Conceptually, *we*-ness is very similar to Aron et al.’s (1992) IOS measure of self-other overlap, which consists of a set of two circles that overlap to different degrees. Indeed, one study found that individuals who used more *we*-talk when describing their relationship reported greater perceived overlap with their partner (Agnew et al., 1998). In addition, individuals who reported greater self-other overlap with a close friend showed a similar neural response when their friend experienced threat as when they themselves experienced threat, suggesting that *we*-ness with a close other may also be detected at the neural level.

Both interpersonal coping models reviewed in this paper propose some aspect of relationship functioning as an antecedent to dyadic or communal coping processes in couples (Rentscher, 2017; Helgeson et al., 2018). Specifically, Bodenmann (1995) outlined that partners may be motivated to engage in dyadic coping when they are high in marital satisfaction, a sense of togetherness, and/or goals for the future of the relationship. Likewise, Lyons et al. (1998) posited that communal coping is more likely to occur in relationships that are characterized by a high degree of closeness, and Helgeson et al. (2018) proposed relationship quality as one key antecedent to communal coping. To date, several studies have found concurrent associations between relationship quality and communal coping (Rohrbaugh et al., 2008; Robbins et al., 2013; Van Vleet and Helgeson, 2016), and one study found that higher marital satisfaction was associated with greater dyadic coping concurrently and over a five-year period (Bodenmann, 2005). Furthermore, recent studies of communal coping during couple-focused interventions for health problems have found that lower spousal pretreatment relationship distress was associated with greater patient *we*-talk during the first and mid-treatment therapy sessions (Hallgren and McCrady, 2016) and greater couple *we*-talk during a pretreatment conflict discussion was associated with larger within-session increases in *we*-talk among spouses following therapist implementation of techniques that aimed to promote communal coping (Rentscher, 2017). These findings suggest that relational *we*-ness may prime couples to engage in communal coping more readily in a therapeutic context, or be more sensitive to the effects of a communal coping intervention. In addition, these studies have employed a variety of methods to assess relational *we*-ness, including self-report (e.g., IOS), language, and observational measures.

In light of this emerging literature, I propose relational *we*-ness as a unique aspect of relationship quality and antecedent of communal coping in the context of health-related and other stressors. Specifically, it follows from Lyons et al.’s conceptualization of communal coping that a high level of *we*-ness, or sense of togetherness as a couple, is likely to influence partners’ appraisals of stressors as shared rather than individual burdens and activate a process of collaborative action to address the problem in ways that other aspects of relationship quality such as satisfaction or intimacy may not. Future research will therefore benefit from empirical investigation of relational *we*-ness as a key source of relational strength and antecedent of communal and dyadic coping processes.

### Conclusions

The last 20 years have seen the emergence of two prominent interpersonal perspectives on stress and coping that account for the importance of social relationships in the coping process: the STM of dyadic coping and communal coping. This article outlined these two perspectives, highlighting their points of convergence and divergence, and proposing that one difference between the models is that communal coping involves a more explicit focus on a communal or shared appraisal process. Over the last decade, researchers have developed several methods for assessing communal coping, including self-report, language use, and behavioral observation across laboratory, intervention, and real-world settings. This growing body of research has linked communal coping—particularly by the spouse—to better relationship functioning, adjustment to chronic illness, physical health outcomes, and health behavior change in couples coping with health problems. On account of this research, I proposed the utility of incorporating measurement of shared appraisal into future research on dyadic coping with stress, because of its potential to impact health through its influence on primary and secondary stress appraisal processes and physiological stress response systems. As directions for future research, I propose an integrated conceptual model of relational *we-*ness, communal coping, and health, whereby in the face of stress, relational *we-*ness translates into a communal approach to coping (i.e., shared appraisal, collaborative action) that influences biological and behavioral pathways to affect health outcomes (Figure 1). This model has the potential to advance research on communal coping process and measurement, improve our understanding of the pathways through which communal appraisal and collaboration may affect health, and inform intervention development by identifying couples that may be at increased risk for stress-related health declines (e.g., those low in relational *we*-ness) and may therefore benefit from targeted interventions.

## Author Contributions

KR is the sole author and contributor to the work in this manuscript.

### Conflict of Interest Statement

The author declares that the research was conducted in the absence of any commercial or financial relationships that could be construed as a potential conflict of interest.
